# Can you have multiple attentional templates? Large-scale replications of Van Moorselaar, Theeuwes, and Olivers (2014) and Hollingworth and Beck (2016)

**DOI:** 10.3758/s13414-019-01791-8

**Published:** 2019-07-15

**Authors:** Marcella Frătescu, Dirk Van Moorselaar, Sebastiaan Mathôt

**Affiliations:** 1 Department of Experimental Psychology, University of Groningen, Grote Kruisstraat 2/1, 9712TS, Groningen, The Netherlands; 2 Department of Brain and Cognition, University of Amsterdam, Amsterdam, Netherlands

**Keywords:** Visual working memory, Memory capture, Multiple-state account, Accessory, Attentional template, Single-item-template; Multiple-item-template

## Abstract

Stimuli that resemble the content of visual working memory (VWM) capture attention. However, theories disagree on how many VWM items can bias attention simultaneously. According to some theories, there is a distinction between active and passive states in VWM, such that only items held in an active state can bias attention. The single-item-template hypothesis holds that only one item can be in an active state and thus can bias attention. In contrast, the multiple-item-template hypothesis posits that multiple VWM items can be in an activate state simultaneously, and thus can bias attention. Recently, Van Moorselaar, Theeuwes, and Olivers (*Journal of Experimental Psychology: Human Perception and Performance*, *40*(4):1450, 2014) and Hollingworth and Beck (*Journal of Experimental Psychology: Human Perception and Performance*, *42*(7):911–917, 2016) tested these accounts, but obtained seemingly contradictory results. Van Moorselaar et al. (2014) found that a distractor in a visual-search task captured attention more when it matched the content of VWM (memory-driven capture). Crucially, memory-driven capture disappeared when more than one item was held in VWM, in line with the single-item-template hypothesis. In contrast, Hollingworth and Beck (2016) found memory-driven capture even when multiple items were kept in VWM, in line with the multiple-item-template hypothesis. Considering these mixed results, we replicated both studies with a larger sample, and found that all key results are reliable. It is unclear to what extent these divergent results are due to paradigm differences between the studies. We conclude that is crucial to our understanding of VWM to determine the boundary conditions under which memory-driven capture occurs.

Regardless of whether you search for a keychain or a mustard bottle, an internal representation of the target object is stored in visual working memory (VWM; Bundesen, 1990; Bundesen, Habekost, & Kyllingsbæk, 2005; Desimone & Duncan, 1995). Such *attentional templates* play a crucial role in optimizing search by means of top-down influences. Behavioral studies showed this by having participants maintain a color in memory while performing a visual-search task. These studies generally find that search is slower when the display contains a colored distractor that matched the remembered color, relative to a color-unrelated distractor (Olivers, Meijer, & Theeuwes, 2006; Soto & Humphreys, 2007, 2008). This *memory-driven attentional capture* suggests that visual working memory and visual attention share content-specific representations (Olivers et al., 2006), which leads to the prediction that items interfere with visual search whenever they match the content of VWM. Put differently, all yellow bottles in the fridge will capture your attention when you search for the mustard.

Theories differ in the number of items that they postulate can take the role of attentional templates. Olivers and colleagues (2011) proposed a single-item-template (SIT) hypothesis. According to this hypothesis, there are two distinct states within VWM: an *active state*, in which an item takes the role of attentional template and consequently biases attention toward task-relevant input; and a *passive state*, in which items are stored in VWM but do not interact with visual sensory input. Crucially, according to the SIT hypothesis, only a single item can be in an active state at a given time. As such, although it is possible to store multiple items in VWM, only one item would affect visual search. In a later study (Van Moorselaar, Theeuwes, & Olivers, 2014), an additional assumption was added to the SIT hypothesis: When multiple items are held in VWM, competition between the items would arise, preventing *any* item from reaching the state of attentional template, eliminating the effect of memory-driven attentional capture (cf. Van Moorselaar et al., 2014). In sum, the single-item-template (SIT) hypothesis allows one item only to be raised to the level of an active template, and by virtue of being in that state, to bias visual search toward items that resembled this template.

In a study that supports the SIT hypothesis, Dombrowe, Donk, and Olivers (2011) assessed eye movements in an experiment in which participants made a sequence of two eye movements to two colored target objects. In one condition, both targets had the same color, thus requiring only a single attentional template; in another condition, the targets had two different colors, thus requiring two attentional templates. Crucially, Dombrowe et al. (2011) found that the interval between the end of the first eye movement and the initiation of the second eye movement was delayed by about 250–300 ms when the targets had different colors. The authors interpreted this delay as a cost associated with switching attentional templates, and thus as evidence that only one attentional template can be active at a time.

However, switch costs as reported by Dombrowe et al. (2011) are not always found (e.g., Beck, Hollingworth, & Luck, 2012). To understand why (not), Ort, Fahrenfort, and Olivers (2017) conducted an experiment in which participants were, in one condition, forced to stay with, or switch between, target templates (cf. Dombrowe et al., 2011), while in another condition, participants could freely choose to stay or switch. In the free-choice condition, participants first made an eye movement toward one of two VWM items; next, for the second eye movement, both VWM items were shown as potential targets, and participants could freely make an eye movement toward either one. This allowed for proactive control (Braver, 2012), such that participants could already switch between target templates in preparation of the second eye movement. Crucially, Ort et al. (2017) showed that there was a cost for switching between target templates, as predicted by the SIT hypothesis, but that this cost is only observed when participants are forced to switch between target templates, and not when participants can freely select an item that matches whatever template is currently active.

Van Moorselaar et al. (2014) further tested the SIT hypothesis by having participants maintain a color in memory and simultaneously perform a visual-search task, a paradigm partially adopted from Olivers et al. (2006). Participants were first presented with a memory task, where they had to remember one, two, three, or four colored items located at one of four possible locations on the screen. Next, participants performed a visual-search task in which they searched for a target among distractors and indicated in which direction the target’s line segment was tilted. Notably, one of the distractors (if one was present) had a particular color: different or identical to one of the colors presented in the memory task. Van Moorselaar et al. (2014) found that memory-matching distractors capture attention, but, crucially, only when participants kept a single item in memory. The additional effect of memory-driven capture disappeared at higher memory loads, such that it became comparable to the regular capture effect of a colored distractor that is unrelated to VWM. This finding is unexpected, even under the SIT hypothesis: If participants maintain two items in VWM, but only one of these serves as an attentional template, then there’s still a 50% chance of the template item being a match to the colored distractor. On average, this should result in a reduced, but nonzero, memory-driven capture effect. Therefore, to explain their results, Van Moorselaar et al. (2014) suggested, as an additional assumption to the SIT hypothesis, that multiple VWM items competed with each other, and that as a result none of the items became an attentional template.

However, not all studies have found support for the SIT hypothesis (e.g., Adamo, Pun, Pratt, & Ferber, 2008; Bahle, Beck, & Hollingworth, 2018). The question of whether multiple items can be stored in an active state in VWM was assessed by Beck, Hollingworth, and Luck (2012) who instructed participants to search for targets of two colors simultaneously versus one color at a time (i.e., sequentially) in a display filled with targets and distractors. Participants frequently alternated between differently colored targets (Beck et al., 2012). More importantly, in contrast to when participants fixated the same target color twice in a row, no switch costs were revealed prior to shifting between two differing target colors. Based on this lack of switch costs, the authors concluded that maintaining two active templates in VWM is possible, a view termed the multiple-item-template (MIT) hypothesis. (However, as argued by Ort et al., 2017, and as discussed above, the lack of switch costs might also be because, in this paradigm, participants could freely choose whether to stay with, or switch between, target templates.)

The MIT hypothesis is based on the view that VWM representations are stored in a distributed manner across sensory, parietal, and prefrontal networks (Christophel, Klink, Spitzer, Roelfsema, & Haynes, 2017). As such, Kristjánsson and Kristjánsson (2018) argue it unlikely for an attentional bottleneck to limit guidance to a single item, since that would most likely require a singular, VWM-specific neural mechanism. Phrased differently, if multiple VWM items are retained in sensory networks, why would only one of these items be capable of guiding attention? Grubert, Carlisle, and Eimer (2016) indeed showed that maintaining two VWM items triggers attention-related components, associated with activity in early visual cortex (Luck & Hillyard, 1994), as measured through electroencephalography (EEG). Specifically, when a stimulus that matches one of two VWM items is presented, an N2pc component is triggered, which is believed to reflect orienting of attention (Luck & Hillyard, 1994). However, the N2pc that was triggered when two items were held in VWM was smaller in amplitude and emerged a bit later in time (about 30 ms) compared with when only one item was held VWM. This suggests that multiple VWM items can guide attention, though in a less effective manner as the number of VWM items increases (Grubert et al., 2016).

Recently, Hollingworth and Beck (2016; see also Beck & Hollingworth, 2017) tested the MIT hypothesis in a paradigm where participants had to keep either one or two colors in VWM. Next, and very similar to the paradigm used by Van Moorselaar et al. (2014), a visual-search task followed, in which participants indicated the orientation of a target among seven other distractors. The type of distractor was manipulated, with the array containing either only uncolored distractors; two distractors, both having colors unrelated to items held in VWM; two distractors, one of which had the same color as (one of the) items held in VWM; or two distractors, both matching the color of the items held in VWM. Lastly, participants were tested on one of the items in the memory task. Crucially, Hollingworth and Beck (2016) found memory-driven capture, even when participants held two stimuli in working memory. More so, when participants held two colors in VWM and both distractors matched these colors, there was more memory-driven capture than when participants held one color in working memory and one of the distractors matched this color. This finding is important, because it suggests that two colors can serve as attentional templates at the same time. This finding also contrast sharply with the study by Van Moorselaar et al. (2014), who did not find memory-driven capture when participants held two colors in VWM.

Here, we present a replication of two studies: experiment one from Van Moorselaar et al. (2014), and the gap-location task from Hollingworth and Beck (2016). Because previous studies on memory-driven capture have produced mixed findings, and were generally based on small samples, we feel that it is important to replicate two of the studies supporting different hypotheses regarding the interaction between attention and VWM. We replicated Van Moorselaar et al.’ (2014) first (singleton-shape task) experiment using the original procedure. However, only conditions using Memory Load 1 and 2 were tested, because the crucial comparison is between these two conditions. To foresee the results, the memory-capture effect was observed at Memory Load 1, but not at Memory Load 2, as reported by Van Moorselaar et al. (2014). The replication of Hollingworth and Beck’s (2016) gap-location task also used their original procedure, save for one modification: we only tested a memory load of two items. Again, we replicated the memory-driven-capture effect at Load 2 with both one and two distractors matching the color of the VWM items (i.e., partial and full capture).

## Experiment 1

The aim of Experiment 1 was to replicate the main finding of Van Moorselaar et al. (2014, Experiment 1) that memory-driven capture occurs at a memory load of one item, but disappears at a memory load of two items.

### Method

Experimental scripts, data, and analysis scripts can be found on the Open Science Framework (https://osf.io/pwhkc/). The experiment was conducted as part of a course. Twenty students, who were enrolled in this course, tested a total of 66 participants, who participated voluntarily and did not receive compensation. The experiment was approved by the Ethics Review Board of the Department of Psychology of the University of Groningen (#17364-O). Stimuli were generated with OpenSesame 3.1 (Mathôt, Schreij, & Theeuwes, 2012) using the PsychoPy backend (Peirce, 2007). Students downloaded this software on their own computers, recruited participants themselves, and performed testing in any environment that they considered suitable. (That is, the data were collected “in the wild,” and not in a traditional laboratory setting.) Participants provided verbal informed consent prior to participation and were allowed to (and frequently did) abort the experiment whenever they wanted.

**Fig. 1:**
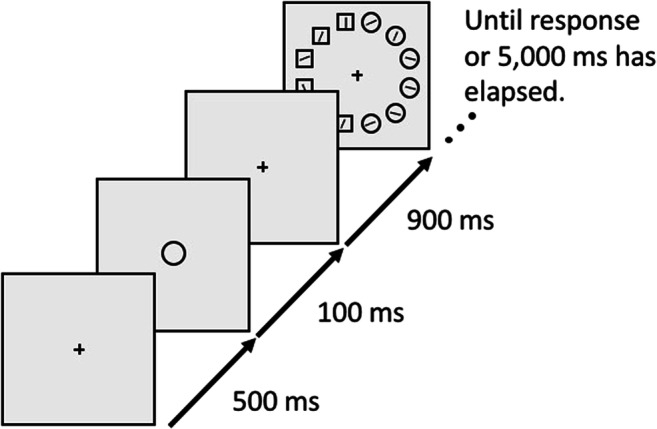
A schematic example trial of Experiment 1. This example demonstrates a Load 2 trial, because the participant needs to remember two colors. For visualization, all distractor conditions are shown for the search display. However, only one distractor condition occurred on each trial. (Color figure online)

The task, illustrated in Fig. 1, was closely modeled after Experiment 1 from Van Moorselaar et al. (2014), and implemented by the original author of that study (D.M.). Each trial started with a 750-ms black fixation cross. Next, a 1,000-ms memory display was shown with one (Load 1) or two (Load 2) colored squares placed at a random subset of four possible locations centered on the intercardinal axes. All stimuli were presented at a distance of 200 pixels from the central fixation point, and were about 50 pixels in diameter. Memory colors were randomly selected from five color categories (red, green, yellow, blue, and purple); each color category had nine different exemplars with different combinations of hue and chroma, but all with a similar brightness (except for yellow, which was overall brighter to make it appear less brown). The memory display was followed by a 1,250 ms fixation display, followed in turn by the search display.

Each search display consisted of one white diamond-shaped target and seven white disk-shaped distractors, all with a leftward or rightward-tilted white line segment in their center. Stimuli were presented on an imaginary circle around the central fixation dot, also with a 200 pixel radius, such that the location of the distractors at the intercardinal locations matched the four possible memory locations. The location of the target was randomly selected, with the restriction that it was never displayed on one of the memory locations of that trial. Participants used the arrow buttons on the keyboard to indicate as quickly and accurately as possible whether the target line segment was tilted leftward or rightward from a vertical orientation. In case of an incorrect response, the 250-ms fixation display that followed the search display was replaced by the word “incorrect” displayed in red at the center of the screen.

On 75% of the trials, one of the distractors, located at one of the memory locations of that trial, was replaced by a colored disk with a color selected from the memory color pool. There were four distractor conditions, randomly mixed within blocks. In the unrelated-distractor condition, the color category of the distractor was different from the color categories present in the memory display. In the color-related/location-match-distractor condition, the distractor color matched the memory color that was presented on that location in the preceding memory display. In the color-related/location-mismatch-distractor condition, the distractor color also matched one of the memory colors, but the colored distractor now appeared at the position of one of the other memory colors. (In the case of Load 1, this meant that the distractor was placed on an empty memory location.) Finally, in the no-distractor condition, there was no colored distractor present in the search display.

Although the colored distractor could be shown at all four possible memory locations, throughout the entire experiment, one of these locations had a higher probability (66.7%), and this location was varied between subjects. Trials on which the color distractor appeared at these highly probable locations were called *suppressed*. The other trials were called *non-suppressed*. This *suppression* factor was included to replicate a study by Wang and Theeuwes (2018) on statistical learning of distractor suppression. The results of this (successful) replication can be found on the Open Science Framework.^1^

The trial sequence ended with a change-detection memory test, in which colored squares were presented on the memory locations of that trial. On half of the trials these squares were identical to the memory display, whereas on the other half of the trials the color of one of the squares changed to another exemplar from the same color category. A one-pixel-thick line cued one square, and participants were instructed to indicate with a key press whether the cued square changed or was identical to the memory item (“C” for change and “N” for no change).

All participants completed 15 practice trials and 10 experimental blocks of 48 trials each. Within an experimental block, each distractor condition was present six times for each of the memory loads, randomly mixed, resulting in 30 observations per condition. In between blocks, participants were encouraged to take a short break while they received feedback on reaction times (RTs) and accuracy in the search task, and their accuracy in the memory task.

### Results

#### Trial and participant exclusion criteria

Exclusion criteria were based on, but not identical to, Van Moorselaar et al. (2014). Data analysis was performed by S.M., based on clarifications by the original author (D.M.).

First, all trials with a reaction time of less than 200 ms or more than 5,000 ms were excluded. Next, for each participant, trials on which the reaction time (RT) deviated more than 2.5 standard deviations from that participant’s mean correct RT were excluded. Next, participants were excluded if their mean RT deviated more than 2.5 standard deviations from the grand mean RT, or their performance on the memory task was not significantly different from chance (*p* ≥ .05), as determined by a chi-squared (χ^2^) test. (Two participants performed far below chance on the memory task. These were not excluded based on the assumption that they had inadvertently reversed the response rule, but had otherwise performed the task correctly.)

Fifty-six participants (of 66) and 26,180 trials (of 32,670) remained for further analysis. All results described below are robust to different trimming procedures, including the one used in Van Moorselaar et al. (2014).

#### Effects of memory load and distractor condition on search performance

**Fig. 2:**
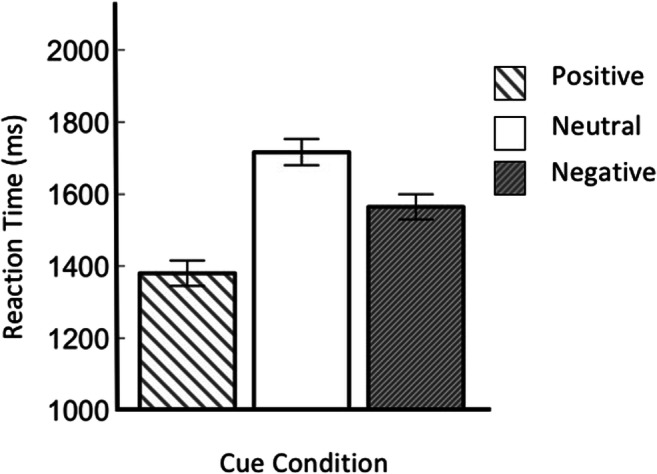
Main results of Experiment 1. Mean response time as a function of distractor condition (*x*-axis) and memory load (different lines). (Color figure online)

We first conducted a Bayesian repeated-measures analysis of variance (ANOVA), with per-participant mean RT as the dependent variable, and distractor condition and memory load as the independent variables. We used the inclusion Bayes factor based on matched models (“Baws factor”) to assess the evidence for individual effects (Mathôt, 2017). The labels for strength of evidence are those proposed by Jeffreys (1961; as cited in Wetzels et al., 2011). All tests were conducted in JASP 0.8 with the default Cauchy prior (center = 0; *r* = .707). Figure 2.

There was decisive evidence for an effect of distractor condition (BF_10_ = 6.00 × 10^53^), reflecting that participants were overall fastest in the no-distractor condition, slower in the unrelated-distractor condition, and slowest of all in the two related-distractor conditions. And there was decisive evidence for an effect of memory load (BF_10_ = 589.48), reflecting that participants were overall slower on Load 1 than on Load 2 trials. Crucially, there was substantial evidence for a Distractor Condition × Memory Load interaction (BF_10_ = 7.99), reflecting that there was a difference between the related-distractor conditions and the unrelated-distractor condition on Load 1 trials, but not on Load 2 trials (tested in more detail below).

To further characterize the crucial Distractor Condition × Memory Load interaction, which was also reported by Van Moorselaar et al. (2014), we conducted a set of two-tailed Bayesian paired-samples *t* tests. In the Load 1 condition, there was strong evidence for higher RTs on color-related/location-match-distractor trials than on unrelated-distractor trials (BF_10_ = 36.51), and decisive evidence for higher RTs on color-related/location-mismatch-distractor trials than on unrelated-distractor trials (BF_10_ = 1.19 × 10^5^). In contrast, in the Load 2 condition, there was substantial evidence for similar RTs on color-related/location-match-distractor trials and on unrelated-distractor trials (BF_10_ = 0.163), and substantial evidence for similar RTs on color-related/location-mismatch-distractor trials and on unrelated-distractor trials (BF_10_ = 0.117). In summary, and consistent with Van Moorselaar et al. (2014), we found memory-driven attentional capture only for a memory load of one item, and not at all for a memory load of two items.

**Fig. 3:**
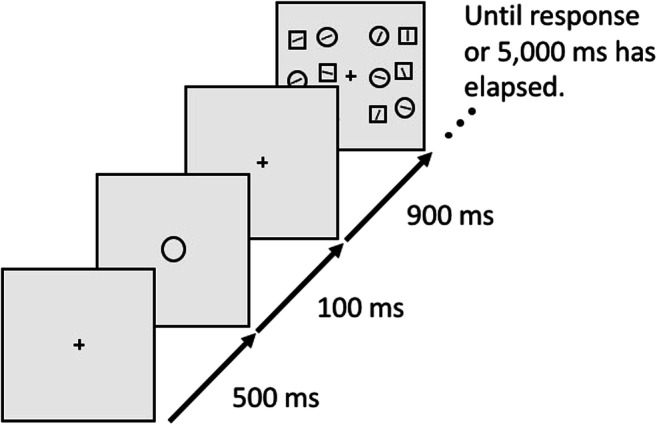
Individual results for Experiment 1. The strength of memory-driven attentional capture for individual participants on Load 1 and Load 2 trials. Memory-driven attentional capture is the difference in response time between the unrelated distractor condition and the average of the color related/location mismatch and color related/location match conditions. Data points are rank-ordered by effect size, separately for Load 1 and Load 2. (Color figure online)

As shown in Fig. 3, this pattern was fairly consistent across participants, such that the majority (43 of 56) of the participants showed memory-driven capture in the Load 1 condition, and half (28 of 56) of the participants showed memory-driven capture in the Load 2 condition, as would be expected by chance.

#### Effect of memory load on memory performance

Finally, as shown by a Bayesian paired-samples two-tailed *t* test (BF), there was decisive evidence for higher memory accuracy on Load 1 (76%) than on Load 2 (67%) trials (BF_10_ = 3.52 × 10^20^).

### Discussion

We successfully replicated the key finding of Van Moorselaar et al. (2014): In their paradigm, memory-driven capture reliably occurs with a memory load of one item, but is completely abolished with a memory load of two items.

## Experiment 2

The aim of Experiment 2 was to replicate the main finding of the gap-location task of Hollingworth and Beck (2016), which showed, in contrast to Van Moorselaar et al. (2014) and our own Experiment 1, that memory-driven capture does occur at a memory load of two items. A detailed preregistration of this experiment is available on the Open Science Framework (https://osf.io/9k26n/).

The overall approach of Experiment 2 was similar to that of Experiment 1. Differences are described below.

**Fig. 4:**
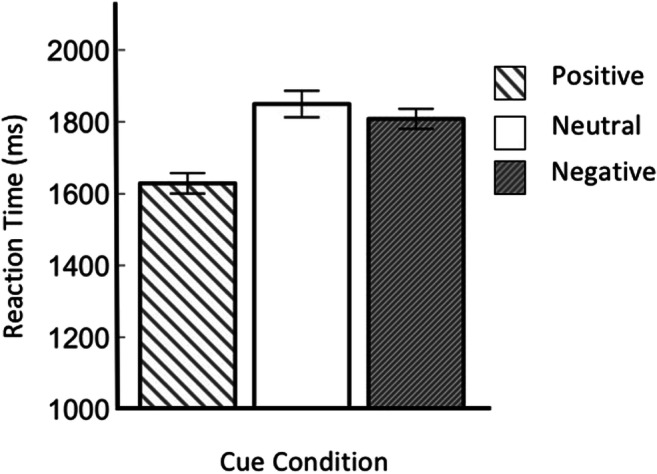
A schematic example trial of Experiment 2. For visualization, all distractor conditions are shown for the search display. (Color figure online)

Students tested a total of 81 participants as part of a course. Stimuli were generated with OpenSesame 3.2 (Mathôt et al., 2012) using the Expyriment backend (Krause & Lindemann, 2014). The task, illustrated in Fig. 4, was closely modeled after the gap-location task used by Hollingworth and Beck (2016), based on the original script used in that study, and reimplemented by two authors of the current paper (M.F. and S.M.). Each trial started with a 500-ms white fixation dot. Next, a 250-ms memory display appeared, containing two colored squares placed at opposite sides of an imaginary circle around a central fixation dot, with a 128 pixel radius. The memory display was followed by a 750-ms fixation display, followed in turn by the search display.

The search display consisted of eight square-like stimuli, one target and seven distractors, that were open on one side. The target was open on the left or right side. The distractors were open on the upper or lower side. Stimuli were shown on an imaginary circle around the central fixation dot, with a radius of 200 pixels. Participants used the arrow buttons on the keyboard to indicate as quickly and accurately as possible whether the target was open on the left or the right side.

There were four distractor conditions, randomly mixed within blocks, but occurring with different frequencies. In the color-unrelated (20% of trials) condition, two of the distractors were colored (i.e., not white), but their color category differed from the color categories present in the memory display. In the Match-1 (20% of trials) condition, two colored distractors were shown, one of which matched one of the color categories of the memory display, and one of which did not. In the Match-2 (20% of trials) condition, two colored distractors were shown, each matching one of the color categories of the memory display. The color match could be exact (e.g., the same shade of green) or categorical (e.g., a slightly different shade of green). Finally, in the no-distractor (41% of trials) condition, there were no colored distractors present in the search display.

The trial sequence ended with a color-selection memory test, in which two colored squares were shown side by side. One of the squares exactly matched one of the colors of the memory display; the other square was a categorical match. The squares were shown at the same location as the original color had been shown during the memory display. Participants indicated which of the two squares was an exact match with the memory display by pressing the left or right arrow key. After an incorrect response, the word “incorrect” was shown in red for 300 ms. Finally, after a delay of 300 ms, participants were asked to press the space bar to start the next trial.

All participants first completed 10 practice trials that contained only the memory task. Next, participants completed 12 practice trials that contained both the memory task and search task. Next, they completed three experimental blocks of 51 trials each. In between blocks, participants were encouraged to take a short break.

### Results

We used the same exclusion criteria and analyses as in Experiment 1.^2^ Fifty-six participants (of 81) and 8,290 trials (of 14,175) remained for further analysis.

**Fig. 5:**
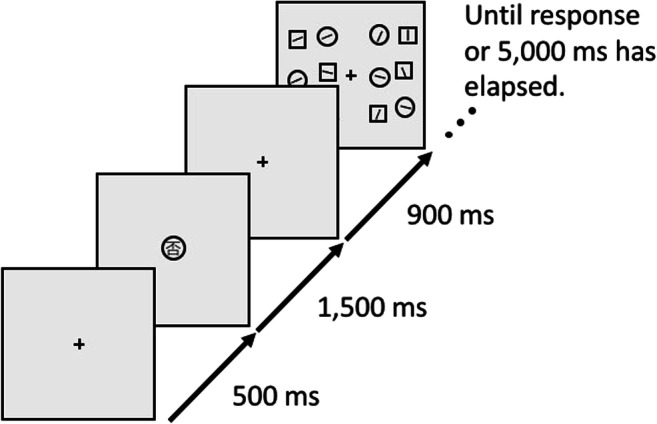
Main results of Experiment 2. Mean response time as a function of distractor condition

We first conducted a Bayesian repeated-measures ANOVA, with per-participant mean RT as the dependent variable, and distractor condition as the independent variable (see Fig. 5). There was decisive evidence for an effect of distractor condition (BF_10_ = 7.26 × 10^9^). To characterize this effect, which was also reported by Hollingworth and Beck (2016), we conducted a set of two-tailed Bayesian paired-samples *t* tests. There was decisive evidence for higher RTs on Match-2 trials than on Match-1 trials (BF_10_ = 158.46). There was substantial evidence for higher RTs on Match-1 trials than on color-unrelated trials (BF_10_ = 3.86). Finally, there was strong evidence for higher RTs on color-unrelated trials than on no-distractor trials (BF_10_ = 15.98).

**Fig. 6:**
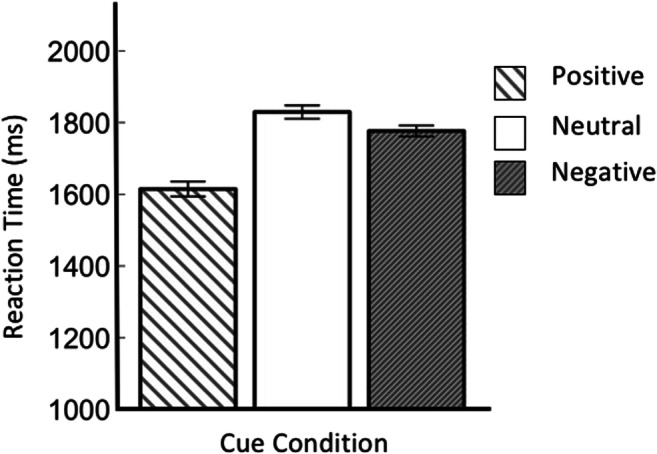
Individual results for Experiment 2. The difference in response time between Match-2 and Match-1 trials (blue) and Match-1 and Color Unrelated trials (red). Data points are rank ordered by effect size, separately for the two contrasts. (Color figure online)

As shown in Fig. 6, this pattern was fairly consistent across participants, such that the majority of participants (42 of 56) showed stronger memory-driven capture on Match-2 than on Match-1 trials, and a majority of participants (35 of 56) also showed stronger capture on Match-1 than on no-distractor trials.

### Discussion

We successfully replicated the key finding of Hollingworth and Beck (2016): In their gap-location task, memory-driven capture reliably occurs with a memory load of two items. And the strength of memory-driven capture is stronger when two distractors match the memorized colors, compared with when only one distractor matches a memorized color.

## Data quality

The current data were collected in an uncontrolled setting by students as part of an undergraduate biological psychology course. To ensure that the quality of the data is comparable to that of data collected in a traditional laboratory setting, we determined the split-half reliability of the response times in both experiments and compared this to two (unpublished) data sets that we recently collected in a traditional laboratory setting, using a very similar combined working-memory/visual-search task. We randomly split the data into two equally sized subsets, and then determined the correlation between the mean per-participant response times in both subsets as a measure of split-half reliability. We repeated this 100 times to determine the mean *r* and the 95% confidence interval. We found split-half reliabilities of *r* = .995 [.993, .997] (Experiment 1) and *r* = .976 [.966, .985] (Experiment 2) for our current wild-type data. Our comparison data sets had split-half reliabilities of *r* = .946 [.914, .970] and *r* = .983 [.974, .990]. In other words, the split-half reliability of our current wild-type data is comparable to that of laboratory data.

## General discussion

The current study successfully replicated two experiments: one by Van Moorselaar et al. (2014) and the other by Hollingworth and Beck (2016). First, in replication of Van Moorselaar et al. (2014), memory-driven attentional capture was observed when participants kept one item in VWM, an effect well established in the attentional/VWM literature (e.g., Soto & Humphreys, 2007; Soto, Humphreys, & Rotshtein, 2007). As such, a distractor that shared the color of the item held in VWM attracted more attention relative to a distractor that did not share such VWM feature content. Importantly, this effect disappeared at Load 2; in this case, memory-driven attentional capture was equivalent to capture by an unrelated distractor. These results provide support for Olivers and colleagues’ (2011) version of the SIT hypothesis, where only one item can take the role of an attentional template, while competition between VWM items will result in none of the items becoming attentional templates.

The replication of Hollingworth and Beck’s (2016) experiment showed a strikingly different pattern. There, memory-driven attentional capture was observed for two items held in VWM when one distractor matched the memory content (partial capture), as well as when both distractors matched the memory content (full capture). It thus seems that it was possible for (at least) one attentional template to be instantiated when two items are held in VWM. The increased time spent on the visual-search task when both distractors, as compared with one distractor, matched the content of VWM, points to the possibility of two attentional templates biasing visual search simultaneously, or a relatively quick switch between equally relevant templates. We did not include a memory load of one, and as such, we cannot compare whether the memory-driven capture for two matching items (given a memory of load of two) is stronger than the memory-driven capture of one matching item (given a memory load of one), which, as argued by Hollingworth and Beck (2016), would be compelling evidence in favor of the MIT hypothesis. However, our results are inconsistent with a version of the SIT hypothesis in which competition between multiple VWM items prevents any of them from becoming an attentional template.

The aforementioned results are puzzling, since both effects are robust, but, at least on the surface, contradictory. Therefore, it is important to mention differences between the two paradigms that could account for discrepancies between results. First, Van Moorselaar et al. (2014) had one colored distractor in the visual-search task, in both the Load 1 and the Load 2 conditions of the experiment. On the other hand, Hollingworth and Beck (2016) had two colored distractors. More so, the timing of the two experiments differed: the memory display in Van Moorselaar et al. (2014) lasted for 1,000 ms (with a subsequent delay of 1,250 ms), while the memory display in Hollingworth and Beck (2016) lasted for 250 ms (with a delay of 700 ms). It has been argued that memory representations are stronger at shorter time intervals (Dombrowe, Olivers, & Donk, 2010). This could explain, at least in part, the observed memory-capture effect at Load 2 in the Hollingworth and Beck (2016) replication compared with Van Moorselaar et al., (2014). However, a recent visual-search paradigm, similar to the current studies, implemented by Chen and Du (2017; see also Fan et al., 2019) showed that memory capture at Load 2 can be found at longer intervals, too. Thus, timing differences between the two studies cannot fully account for the contradictory results. The two experiments also differed in terms of overall response time, with the Hollingworth and Beck (2016) replication yielding slower response times, although it seems unlikely that this can fully account for the qualitatively different results. Lastly, the set of stimuli used by the two experiments differed. Specifically, in the paradigm by Van Moorselaar et al. (2014), the target was defined as the line segment inside a unique shape; therefore, identifying the target was a two-stage process of first identifying the unique shape, and then “zooming in” to identify the orientation of the line segment inside this shape. In contrast, in the paradigm by Hollingworth and Beck (2016), the target was defined as the only shape with an opening to the left or to the right, and could therefore be defined in a single stage. It is conceivable that, in a way that is currently not understood, this seemingly trivial difference could affect the attentional processes involved in identifying the target. In general, although it is unclear which of the differences between these two paradigms could account for the diverging results, the differences are notable and warrant further investigation.

The current replication study is also a compelling demonstration of collecting data in an untraditional, nonlaboratory setting. The experiments were conducted as part of an undergraduate course, and while we carefully instructed students on how to conduct the study, we had no control as to how, when, or in what environment they collected the data. Nevertheless, the reliability of the current data is comparable to data collected in the laboratory. This shows that classroom research (and especially replication) is not only an educational and insightful experience for students, it is also a viable way for researchers to conduct large-scale replication studies that contribute to the current state of the art in the field.

In summary, two different paradigms seemingly provide evidence for two mutually exclusive theories: the SIT hypothesis, which posits that only one item can serve as an attentional template at a time, and in its strongest form, that if multiple items are kept in working memory, then none of them take the role of attentional template; and the MIT hypothesis, which posits that multiple items can serve as attentional templates at a time. A single demonstration of at least one attentional template being active with a memory load of two, as provided by Hollingworth and Beck (2016) and also our successful replication, falsifies a strong version of the SIT hypothesis. On the other hand, a single demonstration of an absence of memory-driven capture with a memory load of two, as provided by Van Moorselaar et al. (2014) and also our successful replication, also suggests that the predictions of the MIT hypothesis do not hold in all situations. This leaves two possibilities: a version of the SIT hypothesis that allows for at least one attentional template, even in situations where multiple items are kept in working memory, or the MIT hypothesis, in which all working-memory items can serve as attentional templates, but do not always do so.

## Footnotes

1 For the purpose of the current manuscript, we note only that suppression did not interact with any of the factors of interest here, and do not consider it further.

2 Because of a bug in the experimental script, the correctness of the visual-search responses was not logged in Experiment 2. Therefore, analyses are performed across all responses, rather than only correct responses, as we had specified in the preregistration. To verify that this did not alter the results, we conducted all analyses for Experiment 1 both with and without correct responses and found that this did not affect any of the results.
